# Urgent need for newborn targeted sequencing screening technology in Shandong Province, China

**DOI:** 10.1007/s12519-025-00907-5

**Published:** 2025-04-29

**Authors:** Jia-Lin Mu, Meng Sun, Yu-Lin Li, Pan-Pan Li, Hui Zou

**Affiliations:** Jinan Maternal and Child Care Hospital, No.2, Jingsan Road, Jianguo Xiao, Shizhong District, Jinan, China

China’s newborn screening (NBS) system, initiated over four decades ago with dual-disease detection [phenylketonuria (PKU) and congenital hypothyroidism (CH)], has progressively expanded to encompass congenital adrenocortical hyperplasia (CAH) and glucose-6-phosphate dehydrogenase (G6PD) deficiency, achieving nationwide universal coverage. This systematic progression has substantially elevated diagnostic rates for these disorders, enabling timely therapeutic interventions [1]. Over the past two decades, technological innovations—notably the integration of tandem mass spectrometry (MS/MS) platforms in pioneering regions such as Shanghai and Zhejiang—have extended NBS to over 40 inherited metabolic diseases (IMDs). The socioeconomic benefits of MS/MS-based large-scale IMD screening have driven its widespread adoption, with annual screening volumes exceeding tens of millions, thereby enhancing birth cohort health outcomes [2]. Currently, over 100 neonatal screening centers nationwide utilize MS/MS platforms for IMD detection.

Jinan City, Shandong Province, exemplifies progressive IMD screening through its fully subsidized, population-wide NBS program. This study synthesizes epidemiological data from Shandong Province (2023) and Jinan City (2019–2024) to delineate disease prevalence patterns, benchmarked against national and global rates. Our analysis underscores the imperative for implementing next-generation sequencing (NGS)-based genetic screening as a primary NBS modality in Shandong Province.

## Results of screening for newborn inherited metabolic diseases in Shandong Province

We present an analysis of data pertaining to neonatal genetic metabolic disease screening in Shandong Province in 2023. The findings revealed that a total of 470,368 neonatal genetic metabolic disease screenings were conducted in the province utilizing the MS/MS platform, resulting in the diagnosis of 324 patients with IMDs, yielding an overall prevalence rate of 1/1452. Among these patients, 160 had amino acid metabolism disorders (prevalence rate of 1/2940); 111 had organic acid metabolism disorders (prevalence rate of 1/4238); 44 had fatty acid metabolism disorders (prevalence rate of 1/10,690); and 9 had urea cycle disorders (prevalence rate of 1/52,263). The prevalence of the various diseases was determined and the three most prevalent diseases were as follows: hyperphenylalaninemia (including phenylalanine hydroxylase deficiency and tetrahydrobiopterin deficiency, 1/3054), methylmalonic acidemia (1/5469), and primary carnitine deficiency (1/22,398). The prevalence of these diseases is shown in Table 1.

**Table 1:** The number and rate of multiple genetic metabolic diseases by MS/MS in Shandong Province

Number	Disease types	Cases	Rate
1	Hyperphenylalaninemia	154	1/3054
2	Methylmalonic acidemia	86	1/5469
3	Primary carnitine deficiency	21	1/22,398
4	3-Methylcrotonyl-coenzyme A carboxylase deficiency	15	1/31,358
5	Short-chainacyl-coenzyme A dehydrogenase deficiency	14	1/33,598
6	Very long chain acyl-CoA dehydrogenase deficiency	6	1/78,395
7	Citrin deficiency	5	1/94,074
8	Tyrosinemia	4	1/117,592
9	Glutaric acidemia	4	1/117,592
10	Medium chain acyl-CoA dehydrogenase deficiency	3	1/156,789
11	Propionic acidemia	2	1/235,184
12	Ornithine transcarbamylase deficiency	2	1/235,184
13	Isobutyryl-CoA dehydrogenase deficiency	2	1/235,184
14	Isovaleric acidemia	2	1/235,184
15	Hyperhomocysteinemia	2	1/235,184
16	Citrullinemia type I	1	1/470,368
17	Argininosuccinate lyase deficiency	1	1/470,368
Total		324	1/1452

*MS/MS* tandem mass spectrometry

## Results of screening for newborn patients with inherited metabolic diseases in Jinan City

From January 2019 to December 2024, a total of 308,471 newborns were screened in Jinan City, Shandong Province, China, and 178 patients with IMDs were ultimately diagnosed, with a prevalence of 1/1733. Among these patients, 70 were identified as having amino acid metabolism disorders (prevalence of 1/4406); 86 were identified as having organic acid metabolism disorders (prevalence of 1/3586); 20 were identified as having fatty acid metabolic disorders (prevalence of 1/15,423); and 2 were identified as having urea cycle disorders (prevalence of 1/154,235). Table 2 provides a comprehensive overview of the diseases diagnosed as IMDs, along with their respective prevalence rates. Methylmalonic acidemia, a condition that falls within the category of organic acid metabolism disorders, was observed in 79 patients, with a prevalence of 1/3905. Hyperphenylalaninemia, a condition characterized by an accumulation of phenylalanine, hydroxy-tetrahydrobiopterin deficiency, and tetrahydrobiopterin deficiency associated with amino acid metabolism disorders, was identified in 59 patients, with a prevalence of 1/5056. Primary carnitine deficiency among patients with fatty acid metabolism disorders was observed in 12 patients, with a prevalence of 1/25,706.

**Table 2:** The number and rate of multiple genetic metabolic diseases by MS/MS in Jinan City

Number	Disease types	Cases	Rate
1	Methylmalonic acidemia	79	1/3905
2	Hyperphenylalaninemia	61	1/5056
3	Primary carnitine deficiency	12	1/25,706
4	Medium chain acyl-CoA dehydrogenase deficiency	4	1/77,118
5	Hypermethioninemia	4	1/77,118
6	Hepatorenal tyrosinemia	3	1/102,824
7	Isobutyryl-CoA dehydrogenase deficiency	3	1/102,824
8	3-methylcrotonyl-coenzyme A carboxylase deficiency	2	1/154,236
9	Short-chain acyl-coenzyme A dehydrogenase deficiency	2	1/154,236
10	Very long-chain acyl-CoA dehydrogenase deficiency	1	1/308,471
11	Maple syrup urine disease	1	1/308,471
12	Hyperhomocysteinemia	1	1/308,471
13	Argininemia	1	1/308,471
14	Carnitine palmitoyl transferase II deficiency	1	1/308,471
15	Glutaricacidemia type 1	1	1/308,471
16	Multiple acyl-CoA dehydrogenase deficiency	1	1/308,471
17	Citrin deficiency	1	1/308,471
Total		178	1/1,733

*MS/MS* tandem mass spectrometry

Newborn screening is recognized as a vital component of the tertiary prevention and control of birth defects and as an important international measure for the early detection of patients with inherited metabolic disorders. It is well known for its benefits in preventing birth defects, improving population quality, and improving social outcomes [3, 4]. In recent years, as awareness of inherited metabolic diseases has increased worldwide, early identification and intervention in patients with inherited metabolic diseases can greatly improve the prognosis of patients [5, 6]. Moreover, as a reliable epidemiological survey method, newborn screening for inherited metabolic diseases provides important data for improving the diagnosis and treatment of inherited metabolic diseases, improving the relevant social security system and drug development.

A systematic evaluation based on global data on inherited metabolic defects from 1980 to 2017 revealed that the overall prevalence of IMDs in the neonatal period was 1/1965 [7]. The prevalence rates were 1/1015 in Iran [8], 1/2607 in Portugal [9], 1/3065 in Singapore [10], and 1/26,000 in Germany [11]. There is some variability in the prevalence of IMDs across different countries and regions, and a certain correlation with the types of diseases included in disease screening cannot be excluded. According to the results of a survey conducted by the China Neonatal Disease Screening Expert Group in 2018, the prevalence of IMDs in China was 1/3153 and the prevalence of IMDs in southern China (Shanghai, Zhejiang, etc.) was 1/4153 to 1/3795 [1, 12], which is the same as the national prevalence. In this study, the prevalence of IMDs in Shandong Province was reported to be 1/1452, which is significantly higher than the prevalence of IMDs in China and globally, suggesting that this large group of diseases is more prevalent in Shandong Province. In Jinan, the first city in Shandong Province to conduct newborn screening for inherited metabolic diseases, the prevalence of IMDs was 1/1733, which is consistent with the provincial level; but the prevalence of disorders of organic acid metabolism was 1/3586, which is slightly higher than the provincial prevalence (1/4238), suggesting that there is some variability in the disease types among different cities in the same province. For single disease types, hyperphenylalaninemia (including phenylalanine hydroxylase deficiency and tetrahydrobiopterin deficiency) in amino acid metabolism disorders, methylmalonic acidemia in organic acid metabolism disorders, and primary carnitine deficiency in fatty acid metabolism disorders in Shandong Province were the same as the common disease types reported nationwide. However, the prevalence of hyperphenylalaninemia (1/3054) was much higher than the national prevalence (1/10,712), so as the prevalence of methylmalonic acidemia (1/5469) compared to national prevalence (1/15,213). The results of screening for methylmalonic acidemia in Jinan City in particular revealed a high prevalence of the disease of 1/3905 and this great discrepancy in prevalence warrants attention. The pronounced heterogeneity in disease distribution across geographic regions may reflect complex interactions between genetic predispositions and environmental exposures. In a study of methylmalonic acidemia (MMA) carriers in Jinan in 2023, 4.68% of the 6800 newborns included in the study carried MMA-associated pathogenic variants, which is equivalent to 1 in 20, suggesting that the high prevalence of MMA in Jinan has a molecular genetic basis. On the other hand, the prevalence of MMA in Jinan may be underestimated, given that a large proportion of newborns who die without screening may have inherited metabolic disorders and have delayed onset of the disease due to undetected (i.e., false-negative) screening programs. On the basis of this high prevalence and the great harm associated with the development of IMDs, it is important to emphasize the need for newborn screening for inherited metabolic diseases in Shandong Province. To the best of our knowledge, there are currently 16 neonatal screening centers in Shandong Province, of which 10 have a tandem mass spectrometry (MS) screening rate of more than 95%, and the remaining six have a MS screening rate of between 37% and 88%; thus, public education and governmental policy support may be the keys to increasing the screening rate.

By applying MS/MS technology to newborn screening, we recognize the limitations of this technology platform. First, the scope of screening remains constrained. Presently, MS/MS technology is predominantly employed to target numerous metabolic diseases and can detect only diseases that are accompanied by specific markers. However, hundreds of hereditary diseases have been recognized [2, 13]. Second, MS/MS technology can detect the degree of increase or decrease in enzyme activity or related specific markers, which are easily affected by metabolism during pregnancy and the postnatal period, as well as various aspects and factors, such as sample collection, sample transport and poor environments [14, 15]. Finally, in some metabolic diseases, the accumulation of metabolites in the early stages of life does not reach the detectable range and the presence of isomers in some test markers leads to poor reflection of the disease, resulting in false-positive/false-negative results [16, 17]. As a result of these problems, the prevalence of the broad category of IMDs may be higher than we expected. Therefore, there is a need to explore the technical means of newborn screening to find expanded screening tools with high sensitivity and specificity, and to further expand the types of conditions screened while ensuring the reliability of screening. Genetic testing technologies, such as high-throughput sequencing, provide an important basis for confirmatory diagnosis in the diagnosis of diseases (especially rare diseases) and have become rapid diagnostic methods for various types of genetic diseases [13, 18–21]. Genetic testing technology applied to newborn screening can be designed according to the needs of the disease to be screened and the number of genes to be tested to compensate for the shortcomings of biochemical screening, which can help with the rapid diagnosis of screening diseases, improve the efficiency of screening, and expand the types of diseases to be screened [22]. In 2019, a multicenter pilot study of newborn genetic screening in China involving 12 hospitals in 10 provinces and regions was conducted, and the results revealed that gene panel sequencing technology can significantly improve the sensitivity and specificity of newborn screening for inherited metabolic disorders when combined with traditional biochemical screening technology, which has high practical value [23]. In 2022, a guideline on the application of genomic technology in China’s newborn screening program was published by a number of Chinese newborn screening associations, marking entry into the era of genome sequencing in China’s newborn screening program [24]. The study entitled “Genomic sequencing as a first-tier screening test and outcomes of newborn screening” [25], published in 2024, has been widely discussed worldwide. The results of this study provide important data to support the use of genetic screening as a first-tier screening method to improve existing newborn screening programs, which is highly important to clinicians, geneticists, and families in providing better healthcare and counseling services and implies that newborn genetic screening, as a complementary technology to newborn screening programs, has a wide range of strong clinical application prospects.

The prevalence of inborn metabolic disorders (IMDs) in newborns in Shandong Province, China, is significantly higher than the national prevalence. Moreover, newborn screening data from Jinan City, China, revealed a high incidence of amino acid and organic acid metabolism disorders, with the prevalence of methylmalonic acidemia and phenylketonuria significantly higher than the national average. It is, therefore, necessary to implement a comprehensive and wide-coverage newborn metabolic disease screening program in Shandong Province, China. The high molecular genetic prevalence of IMDs in this region renders newborn genetic screening a feasible approach as a primary newborn screening method, with the potential to markedly increase the efficiency of newborn screening in Shandong Province, China.

## Data Availability

All relevant data are within the paper. Original data can be acquired from the corresponding author upon reasonable request.

## References

[1] Jiang H, Yang R, Dong A, Wu B, Zhao Z. Progress of newborn screening in China. J Zhejiang Univ Med Sci. 2023;52:673–82. 10.3724/zdxbyxb-2023-0467 PMC10764191 38115737

[2] Therrell BL, Padilla CD, Borrajo GJC, Khneisser I, Schielen PCJI, Knight-Madden J, et al. Current status of newborn bloodspot screening worldwide 2024: a comprehensive review of recent activities (2020–2023). Int J Neonatal Screen. 2024;10:38. 38920845 10.3390/ijns10020038 PMC11203842

[3] Grosse SD, Dezateux C. Newborn screening for inherited metabolic disease. Lancet. 2007;369:5–6. 17208621 10.1016/S0140-6736(07)60005-1

[4] Pandor A, Eastham J, Beverley C, Chilcott J, Paisley S. Clinical effectiveness and cost-effectiveness of neonatal screening for inborn errors of metabolism using tandem mass spectrometry: a systematic review. Health Technol Assess. 2004;8:1–121. 10.3310/hta8120 14982654

[5] Sun M, Li Y, Li P, Li G, Yan Y, Zou H. Analysis of gene variation and long-term follow-up in children with phenylalanine hydroxylase deficiency diagnosed by newborn screening. J Zhejiang Univ Med Sci. 2023;52:701–6. 10.3724/zdxbyxb-2023-0450 PMC10764183 38105703

[6] Hao L, Ling S, Ding S, Qiu W, Zhang H, Zhang K, et al. Long-term follow-up of Chinese patients with methylmalonic acidemia of the cblC and mut subtypes. Pediatr Res. 2024. 10.1038/s41390-024-03581-x. 39306609 10.1038/s41390-024-03581-x PMC12122358

[7] Waters D, Adeloye D, Woolham D, Wastnedge E, Patel S, Rudan I. Global birth prevalence and mortality from inborn errors of metabolism: a systematic analysis of the evidence. J Glob Health. 2018;8:021102. 30479748 10.7189/jogh.08.021102 PMC6237105

[8] Shakiba M, Yasaei M, Saneifard H, Mosallanejad A, Alaei MR, Kobarfard F, et al. Expanded inherited metabolic diseases screening by tandem mass spectrophotometry: the first report from Iran. Mol Genet Metab Rep. 2024;40:101103. 39006123 10.1016/j.ymgmr.2024.101103 PMC11245937

[9] Gonçalves MM, Marcão A, Sousa C, Nogueira C, Fonseca H, Rocha H, et al. Portuguese neonatal screening program: a cohort study of 18 years using MS/MS. Int J Neonatal Screen. 2024;10:25. 38535129 10.3390/ijns10010025 PMC10971468

[10] Lim JS, Tan ES, John CM, Poh S, Yeo SJ, Ang JS, et al. Inborn error of metabolism (IEM) screening in Singapore by electrospray ionization-tandem mass spectrometry (ESI/MS/MS): an 8 year journey from pilot to current program. Mol Genet Metab. 2014;113:53–61. 25102806 10.1016/j.ymgme.2014.07.018

[11] Maier EM, Mütze U, Janzen N, Steuerwald U, Nennstiel U, Odenwald B, et al. Collaborative evaluation study on 18 candidate diseases for newborn screening in 1.77 million samples. J Inherit Metab Dis. 2023;46:1043–62. 37603033 10.1002/jimd.12671

[12] Zhang X, Ji W, Wang Y, Zhou Z, Guo J, Tian G. Comparative analysis of inherited metabolic diseases in normal newborns and high-risk children: insights from a 10-year study in Shanghai. Clin Chim Acta. 2024;558:117893. 38582244 10.1016/j.cca.2024.117893

[13] Mujamammi AH. Insights into national laboratory newborn screening and future prospects. Medicina. 2022;58:272. 35208595 10.3390/medicina58020272 PMC8879506

[14] Zhou W, Li H, Wang C, Wang X, Gu M. Newborn screening for methylmalonic acidemia in a Chinese population: molecular genetic confirmation and genotype phenotype correlations. Front Genet. 2018;9:726. 30728829 10.3389/fgene.2018.00726 PMC6351470

[15] Li R, Tian L, Gao Q, Guo Y, Li G, Li Y, et al. Establishment of cutoff values for newborn screening of six lysosomal storage disorders by tandem mass spectrometry. Front Pediatr. 2022;10:814461. 35419325 10.3389/fped.2022.814461 PMC8995975

[16] Dijkstra AM, Evers-van Vliet K, Heiner-Fokkema MR, Bodewes FAJA, Bos DK, Zsiros J, et al. A false-negative newborn screen for tyrosinemia type 1-need for re-evaluation of newborn screening with succinylacetone. Int J Neonatal Screen. 2023;9:66. 38132825 10.3390/ijns9040066 PMC10744279

[17] Hashimoto K, Yokokawa M, Yamashita D, Yuge K, Otsubo Y. Spinal muscular atrophy type I with false negative in newborn screening: a case report. Cureus. 2023;15:e42382. 37621829 10.7759/cureus.42382 PMC10445771

[18] Stark Z, Scott RH. Genomic newborn screening for rare diseases. Nat Rev Genet. 2023;24:755–66. 37386126 10.1038/s41576-023-00621-w

[19] Remec ZI, Trebusak Podkrajsek K, Repic Lampret B, Kovac J, Groselj U, Tesovnik T, et al. Next-generation sequencing in newborn screening: a review of current state. Front Genet. 2021;12:662254. 34122514 10.3389/fgene.2021.662254 PMC8188483

[20] Spiekerkoetter U, Bick D, Scott R, Hopkins H, Krones T, Gross ES, et al. Genomic newborn screening: are we entering a new era of screening? J Inherit Metab Dis. 2023;46:778–95. 37403863 10.1002/jimd.12650

[21] Reardon S. Fast genetic sequencing saves newborn lives. Nature. 2014;514:13–4. 25279893 10.1038/514013a

[22] Berg JS, Agrawal PB, Bailey DB Jr, Beggs AH, Brenner SE, Brower AM, et al. Newborn sequencing in genomic medicine and public health. Pediatrics. 2017;139:e20162252. 28096516 10.1542/peds.2016-2252 PMC5260149

[23] Yang RL, Qian GL, Wu DW, Miao JK, Yang X, Wu BQ, et al. A multicenter prospective study of next-generation sequencing-based newborn screening for monogenic genetic diseases in China. World J Pediatr. 2023;19:663–73. 36847978 10.1007/s12519-022-00670-x PMC10258179

[24] Tong F, Wang J, Xiao R, Wu BB, Zou CC, Wu DW, et al. Application of next generation sequencing in the screening of monogenic diseases in China, 2021: a consensus among Chinese newborn screening experts. World J Pediatr. 2022;18:235–42. 35292922 10.1007/s12519-022-00522-8

[25] Chen T, Fan C, Huang Y, Feng J, Zhang Y, Miao J, et al. Genomic sequencing as a first-tier screening test and outcomes of newborn screening. JAMA Netw Open. 2023;6:e2331162. 37656460 10.1001/jamanetworkopen.2023.31162 PMC10474521

